# Safety and anti-tumor activity of lisavanbulin administered as 48-hour infusion in patients with ovarian cancer or recurrent glioblastoma: a phase 2a study

**DOI:** 10.1007/s10637-023-01336-9

**Published:** 2023-02-16

**Authors:** Markus Joerger, Thomas Hundsberger, Simon Haefliger, Roger von Moos, Andreas F. Hottinger, Thomas Kaindl, Marc Engelhardt, Michalina Marszewska, Heidi Lane, Patrick Roth, Anastasios Stathis

**Affiliations:** 1 Department of Hematology/Oncology, Cantonal Hospital St. Gallen, St. Gallen, Switzerland; 2 Swiss Group for Clinical Cancer Research, Bern, Switzerland; 3 Department of Neurology, Cantonal Hospital St. Gallen, St. Gallen, Switzerland; 4 Department of Medical Oncology, Inselspital, Bern University Hospital, University of Bern, Bern, Switzerland; 5 Department of Medical Oncology/Hematology, Cantonal Hospital Graubünden, Chur, Switzerland; 6 Lundin Family Brain Tumor Research Center, Departments of Oncology & Clinical Neurosciences, Lausanne University Hospital & University of Lausanne, Lausanne, Switzerland; 7 Basilea Pharmaceutica International Ltd, Hegenheimermattweg 167b, Allschwil, 4123 Switzerland; 8 Department of Neurology and Brain Tumor Center, University Hospital Zurich and University of Zurich, Zurich, Switzerland; 9 Oncology Institute of Southern Switzerland, EOC, Bellinzona, Switzerland

**Keywords:** Lisavanbulin, Avanbulin, BAL101553, Microtubule-targeting agent, Glioblastoma

## Abstract

*Purpose. *Lisavanbulin (BAL101553) is the prodrug of avanbulin (BAL27862), a microtubule-destabilizing agent. The goal of this study (NCT02895360) was to characterize the safety, tolerability and antitumor activity of lisavanbulin administered as a 48-hour intravenous (IV) infusion at the recommended Phase 2 dose (RP2D) of 70 mg/m^2^. Results from the Phase 1 dose-escalation portion of the study identifying the RP2D have been previously reported. Here, we present the findings from the Phase 2a portion of this study. *Methods. *This multi-center, open-label study included patients with ovarian, fallopian-tube, or primary peritoneal cancer that was either platinum-resistant or refractory (11 patients), or with first recurrence of glioblastoma (12 patients). Lisavanbulin was administered as a 48-hour IV infusion on Days 1, 8, and 15 of a 28-day cycle. *Results. *Lisavanbulin was well tolerated in both patient cohorts. Thirteen patients (56.5%) developed 49 adverse events assessed as related to study treatment. The majority were mild or moderate; four were grade 3/4. Sixteen SAEs were reported in nine patients (39.1%), with none considered related to study treatment. No AEs led to permanent treatment discontinuation. Three patients in the ovarian cancer cohort had stable disease with lesion size reductions after two cycles of treatment; in the glioblastoma cohort, one patient showed partial response with a > 90% glioblastoma area reduction as best response, and one patient had stable disease after eight cycles of treatment. *Conclusion. *This study demonstrated a favorable safety and tolerability profile of 48-hour continuous IV infusion of lisavanbulin in patients with solid extracranial tumors or glioblastoma. Clinicaltrials.gov registration: NCT02895360.

**Supplementary Information:**

The online version contains supplementary material available at 10.1007/s10637-023-01336-9.

## Introduction

Microtubule-targeting agents (MTAs) are among the most effective cytotoxic chemotherapy agents, and affect microtubule dynamics either as microtubule-destabilizers (such as the vinca-alkaloids) or microtubule stabilizers (such as the taxanes and azaepothilone B) [1]. Despite the initial sensitivity of many tumors to tubulin-targeting therapy, the development of primary or acquired resistance remains a major challenge [2, 3]. There is a need for novel microtubule-inhibitory agents that can overcome these resistance mechanisms and improve treatment outcomes.

Lisavanbulin (BAL101553; PubChem CID: 45,259,014) is the water-soluble lysine pro-drug of avanbulin (BAL27862; PubChem CID: 11,176,685) [4, 5]. Avanbulin is a potent inhibitor of tumor cell growth due to its ability to bind to tubulin heterodimers at the colchicine-binding site, thereby inhibiting microtubule assembly and resulting in activation of the spindle assembly checkpoint, an event required for anti-tumor activity [5, 6]. The mechanism of action of lisavanbulin extends beyond its ability to target tumor cell proliferation, also affecting the tumor microenvironment by reducing tumor microvasculature [7, 8].

Avanbulin showed activity in a number of *in vitro* and *in vivo* tumor models resistant to other microtubule-targeting agents [9–12]. One clinical study has been completed (CDI-CS-001), investigating lisavanbulin administered as a 2-hour infusion in patients with solid tumors who had failed standard anti-cancer therapy, or for whom no effective standard therapy was available. The recommended Phase 2 dose (RP2D) of 30 mg/m^2^ in that study was well-tolerated and showed encouraging anti-tumor activity [13]. The observed dose-limiting toxicities were related to lisavanbulin’s effects on the vascular system, which occurred at peak serum concentrations (C_max_) [13]. Conversely, the anti-proliferative effects of lisavanbulin were directly correlated to drug exposure (AUC) [4], suggesting that longer exposure at lower doses would result in maximal efficacy.

Study CDI-CS-003, a single-agent, open-label, Phase 1/2a study performed in two parts, aimed to reconcile these aspects of lisavanbulin’s pharmacokinetics through the use of an extended 48-hour IV infusion to maximize exposure while minimizing vascular toxicity. Part 1 of the study (previously reported) was a Phase 1 dose-escalation portion to determine the maximum tolerated dose (MTD) in patients with advanced/recurrent solid tumors [14] and to assess oral bioavailability. No DLTs were reported at the 30 mg/m^2^ or 45 mg/m^2^ doses. One DLT (grade 3 hypotension) was observed at the 70 mg/m^2^ dose [14]. Results from Part 1 of the study indicated that lisavanbulin was well tolerated at doses up to 70 mg/m^2^, and that the MTD (RP2D) for the 48-hour infusion was 70 mg/m^2^. When administered as a continuous 48 h infusion, higher exposures of the active metabolite avanbulin were achieved (compared to a 2-hour infusion at the RP2D), without significant vascular toxicity [14].

The work reported here is from Part 2 of study CDI-CS-003, a Phase 2a expansion portion to further characterize the safety and tolerability of lisavanbulin at the MTD of 70 mg/m^2^, performed in two parallel cohorts of patients with platinum-resistant/refractory ovarian, fallopian tube, or primary peritoneal cancer or glioblastoma at first recurrence. The selection of patients with ovarian cancer was supported by a complete response seen in a patient with ovarian cancer in Phase 1, the activity of avanbulin in taxane- and vinca-alkaloid-resistant ovarian cancer cell and xenograft models [10, 11], and the utility of microtubule-targeting drugs in this indication.

Patients with glioblastoma were not included in Phase 1, but daily oral administration of lisavanbulin was already under investigation as a monotherapy in patients with recurrent glioblastoma in study CDI-CS-002 (NCT02490800 [15]), and in combination with radiotherapy in patients with newly diagnosed glioblastoma in study CDI-CS-004 (NCT03250299). Lisavanbulin demonstrated excellent brain penetration and significant extension of survival alone or in combination with radiotherapy using a panel of glioblastoma patient-derived xenografts [16].

## Methods

### Study design

This was an open-label, multi-center Phase 1/2a study (NCT02895360) conducted between August 2016 and August 2020; results from the completed Phase 2a portion of the study are reported here. The Independent Ethics Committees of the six study sites, and relevant authorities in Switzerland, approved the study protocol. All patients provided written informed consent prior to study participation.

### Objectives

The objectives of the Phase 2a portion of the study were to evaluate the safety, tolerability, and anti-tumor activity of lisavanbulin when administered as a 48-hour infusion at the RP2D in two patient populations of special interest.

### Patients

The study population comprised adult patients (≥ 18 years) with either histologically-confirmed ovarian, fallopian-tube, or primary peritoneal cancer that was platinum-resistant or refractory (the ‘ovarian cancer cohort’), or with histologically-confirmed glioblastoma at first recurrence (the ‘glioblastoma cohort’). Key inclusion criteria were life expectancy ≥ 12 weeks, acceptable organ and marrow function within 15 days prior to starting study drug, Eastern Cooperative Oncology Group (ECOG) performance status ≤ 1 (the ovarian cancer cohort) or ≤ 2 (the glioblastoma cohort), and at least one site of measurable disease as defined by RECIST version 1.1 (ovarian cancer) or RANO criteria (glioblastoma). Glioblastoma patients who were being treated with steroids had to be on a stable or decreasing dose. Patients were excluded if they had received chemotherapy, radiotherapy, immunotherapy, or any investigational agents within 4 weeks (6 weeks for nitrosoureas or mitomycin C, 12 weeks for radiotherapy of glioblastoma) prior to receiving the study drug. Other exclusion criteria were the presence of peripheral neuropathy ≥ CTCAE grade 2, systolic blood pressure ≥ 140 mmHg or diastolic blood pressure ≥ 90 mmHg, and combination treatment with more than two anti-hypertensive medications. Patients with significant cardiac disease or abnormalities, and those with a history of cerebral hemorrhage, cerebral aneurysm, ischemic stroke, or a history of transient ischemic attack within 24 months prior to screening, were also excluded.

### Study treatment

All patients received lisavanbulin administered as a 48-hour IV infusion at 70 mg/m^2^ on Days 1, 8, and 15 of a 28-day treatment cycle, administered via an elastomeric pump (Baxter pump models 2C4711K or 2C1009KP/2C4009K) using an implantable venous access system. All patients were scheduled to receive two treatment cycles. Those with objective response or stable disease were permitted to continue additional treatment cycles beyond cycle 2, until the occurrence of progressive disease or unacceptable toxicity, or until other discontinuation criteria were met.

### Efficacy assessments

Tumor assessments by radiological examination (computed tomography / magnetic resonance imaging scans) and cancer antigen 125 monitoring (in ovarian cancer patients) were performed at screening and end of study, and within the 7 days prior to completion of every even-numbered cycle, before administration of the next cycle of lisavanbulin. Tumor response was evaluated according to RECIST v1.1 (ovarian cancer) or RANO criteria (glioblastoma). Complete response or partial response had to be present for at least 4 weeks to be assessed as confirmed. Each lesion measured at baseline was to be measured throughout the study by the same method of assessment and the same technique, to facilitate consistent assessments and comparisons. Tumor growth was expressed as the change from baseline in the sum/products of the perpendicular diameters for either the target lesion or all measurable enhancing lesions.

### Safety assessments

Safety and tolerability assessments included the recording of adverse events (AEs; evaluated according to CTCAE version 4.03), serious adverse events (SAEs), physical examinations, vital signs measurements, 12-lead electrocardiogram (ECG) assessments including heart rate, and PR, QRS, and QT intervals, clinical laboratory parameters, pregnancy testing, and ECOG performance status. Transthoracic echocardiography was performed at screening and at the end of the study. Concomitant medications were monitored throughout.

### Statistical analysis

All patients who received at least one partial or complete dose of study drug were included in the full analysis population (FAP), and all of these patients who had at least one post-baseline safety assessment were included in the safety population. Separate efficacy-evaluable populations (EEPs) were defined for both cohorts. The EEPs were (1) all patients with progressive disease who had completed at least cycle 1 and who had at least one on-study tumor assessment or radiological assessment, and (2) patients with stable disease, partial response, or complete response at the end of cycle 2, who had received at least four doses of study drug in the first two cycles. The objective response rate was the proportion of patients responding, i.e., with a best observed objective response of complete or partial response. The disease control rate was the proportion of patients with controlled disease (complete or partial response or stable disease) after two and four treatment cycles, and at the end of treatment. Progression-free survival (PFS) was defined as the interval between the date of first infusion and the earliest date of objective disease progression, investigator-confirmed clinical progression, or death due to any cause. Percentages and 95% confidence intervals (CIs) were calculated. Background, demographic and safety data were analyzed using descriptive statistics or contingency tables. Safety assessments included the frequency of AEs and laboratory abnormalities in the safety population. Statistical analyses were performed using SAS® Version 9.3 or higher (SAS Institute, Cary, North Carolina, USA).

## Results

### Patient demographics and medical history

Twenty-three patients were enrolled at six study sites in Switzerland. There were 11 patients in the ovarian cancer cohort and 12 in the glioblastoma cohort, of whom eight (72.7%) and eight (66.7%), respectively, were included in the EEP. Nine patients in each cohort (81.8% and 75%, respectively) completed the follow-up period. The mean age of the FAP was 61.9 years, and all patients were Caucasian. A summary of the diagnosis and extent of cancer at screening for the FAP is shown in Table 1.

**Table 1:** Summary of diagnosis and extent of cancer at screening (full analysis population)

	Ovarian cancer cohort	Glioblastoma cohort
Number of patients	11	12
Age, median (range), years	62.0 (52 − 82)	57.5 (49 − 73)
**Sex, n (%)**		
Male	0	9 (75)
Female	11 (100)	3 (25)
**Primary active tumor, n (%)**		
Brain	0	12 (100)
Fallopian tube	2 (18.2)	0
Ovary	8 (72.7)	0
Peritoneum	1 (9.1)	0
**Tumor histology/cytology, n (%)**		
Carcinosarcoma	1 (9.1)	0
High-grade serous	9 (81.8)	0
Other	1 (9.1)	0
Glioblastoma	0	12 (100)
**Stage classification, n (%)**		
III	4 (36.4)	NA
IV	7 (63.6)	NA
**ECOG**		
Grade 0	6 (54.5)	6 (50.0)
Grade 1	5 (45.5)	4 (33.3)
Grade 2	0	2 (16.7)
**Prior tumor treatment, n (%)**		
Surgery	8 (72.7)	12 (100)
Radiotherapy	0	12 (100)
**Prior lines of systemic treatment, median (range)**		
Overall	2 (1–4)	1 (1)
Taxane-containing	1 (0–2)	0 (0)

The mean (± SD) extent of exposure to lisavanbulin in the safety population was 84.1 (± 119.1) days. The mean exposure was similar in both cohorts (81.6 days vs. 86.5 days in the ovarian cancer and glioblastoma cohorts, respectively).

Six patients with ovarian cancer and one patient with glioblastoma had at least one dose adjustment. The mean (± SD) overall treatment compliance in the safety population was 98.3% (± 4.6), range 80.3–102.9%. This was similar across both cohorts (98.7% and 97.9% in the ovarian cancer and glioblastoma cohorts, respectively).

### Tolerability and safety

Most patients (22/23 [95.7%]) reported at least one treatment-emergent AE. The most frequently-reported AEs were fatigue (10/23 patients, 43.5%), constipation (8/23, 34.8%), decreased appetite (7/23, 30.4%), and abdominal pain (6/23, 26.1%) (see Online Resource 2). One AE led to a dose reduction, but none led to treatment discontinuation. There were 16 SAEs reported by nine patients (39.1%), none of which were considered related to study treatment. No grade 5 toxicity occurred. Related AEs reported for patients in Phase 2a and in those treated at 70 mg/m^2^ in Phase 1 are summarized in Table 2. Apart from reported AEs, there were no significant changes or trends over time, in laboratory assessments, vital signs, ECG results, or echocardiographic assessments.

**Table 2:** Treatment-emergent related adverse events by system organ class, preferred term and worst severity; safety population treated with lisavanbulin 70 mg/m^2^

System Organ Class / Preferred Term, n (%)	Phase 1		Phase 2a				Total (N = 32)	
	Solid tumors (N = 9)		Ovarian cancer (N = 11)		Glioblastoma (N = 12)			
	Grade 1–2	Grade 3–4	Grade 1–2	Grade 3–4	Grade 1–2	Grade 3–4	Grade 1–2	Grade 3–4
**All adverse events**	6 (67)	2 (22)	6 (55)	3 (27)	3 (25)	1 (8)	15 (47)	6 (19)
**General disorders and administration site conditions**	5 (56)	0	4 (36)	0	1 (8)	0	10 (31)	0
Fatigue	4 (44)	0	4 (36)	0	1 (8)	0	9 (28)	0
Chest pain	0	0	1 (9)	0	0	0	1 (3)	0
Gait disturbance	0	0	1 (9)	0	0	0	1 (3)	0
Pyrexia	1 (11)	0	0	0	0	0	1 (3)	0
**Metabolism and nutrition disorders**	4 (44)	0	3 (27)	0	2 (17)	0	9 (28)	0
Decreased appetite	1 (11)	0	1 (9)	0	1 (8)	0	3 (9)	0
Hypokalaemia	1 (11)	0	1 (9)	0	1 (8)	0	3 (9)	0
Hyponatraemia	2 (22)	0	1 (9)	0	0	0	3 (9)	0
Dehydration	1 (11)	0	1 (9)	0	0	0	2 (6)	0
**Gastrointestinal disorders**	4 (44)	0	3 (27)	0	1 (8)	0	8 (25)	0
Diarrhoea	2 (22)	0	2 (18)	0	0	0	4 (13)	0
Abdominal pain	1 (11)	0	2 (18)	0	0	0	3 (9)	0
Nausea	1 (11)	0	1 (9)	0	1 (8)	0	3 (9)	0
Constipation	0	0	2 (18)	0	0	0	2 (6)	0
Vomiting	1 (11)	0	1 (9)	0	0	0	2 (6)	0
Abdominal distension	1 (11)	0	0	0	0	0	1 (3)	0
Flatulence	0	0	1 (9)	0	0	0	1 (3)	0
**Nervous system disorders**	3 (33)	1 (11)	2 (18)	0	1 (8)	0	6 (19)	1 (3)
Paraesthesia	1 (11)	0	2 (18)	0	0	0	3 (9)	0
Neuropathy peripheral	1 (11) ^*^	1 (11)	1 (9) ^*^	0	0	0	2 (6) ^*^	1 (3)
Dysgeusia	0	0	0	0	1 (8)	0	1 (3)	0
Neuralgia	1 (11)	0	0	0	0	0	1 (3)	0
Presyncope	1 (11)	0	0	0	0	0	1 (3)	0
Somnolence	1 (11)	0	0	0	0	0	1 (3)	0
**Musculoskeletal and connective tissue disorders**	0	0	3 (27)	0	1 (8)	0	4 (13)	0
Muscle spasms	0	0	3 (27)	0	1 (8)	0	4 (13)	0
Arthralgia	0	0	1 (9)	0	0	0	1 (3)	0
Muscle tightness	0	0	1 (9)	0	0	0	1 (3)	0
**Psychiatric disorders**	0	0	3 (27)	0	1 (8)	0	4 (13)	0
Hallucination	0	0	1 (9)	0	1 (8)	0	2 (6)	0
Insomnia	0	0	2 (18)	0	0	0	2 (6)	0
Confusional state	0	0	1 (9)	0	0	0	1 (3)	0
**Investigations**	0	0	0	1 (9)	1 (8)	1 (8)	1 (3)	2 (6)
Troponin T increased	0	0	0	0	2 (17)	0	2 (6)	0
Alanine aminotransferase increased	0	0	1 (9)	0	0	0	1 (3)	0
Aspartate aminotransferase increased	0	0	0	1 (9)	0	0	0	1 (3)
Lymphocyte count decreased	0	0	0	0	0	1 (8)	0	1 (3)
Platelet count decreased	0	0	0	0	1 (8)	0	1 (3)	0
**Vascular disorders**	0	1 (11)	0	2 (18)	0	0	0	3 (9)
Hypertension	0	0	0	2 (18)	0	0	0	2 (6)
Hypotension	0	1 (11)	0	0	0	0	0	1 (3)
**Respiratory, thoracic and mediastinal disorders**	0	0	2 (18)	0	0	0	2 (6)	0
Cough	0	0	1 (9)	0	0	0	1 (3)	0
Dyspnoea	0	0	1 (9)	0	0	0	1 (3)	0
**Eye disorders**	0	0	1 (9)	0	0	0	1 (3)	0
Vision blurred	0	0	1 (9)	0	0	0	1 (3)	0
**Infections and infestations**	1 (11)	0	0	0	0	0	1 (3)	0
Lip infection	1 (11)	0	0	0	0	0	1 (3)	0
**Injury, poisoning and procedural complications**	1 (11)	0	0	0	0	0	1 (3)	0
Vascular access complication	1 (11)	0	0	0	0	0	1 (3)	0
**Skin and subcutaneous tissue disorders**	1 (11)	0	0	0	0	0	1 (3)	0
Hyperhidrosis	1 (11)	0	0	0	0	0	1 (3)	0
Preferred terms (PTs) are coded according to MedDRA Version 19.0. ‘Related’ means possibly or probably related. A patient with multiple events within a PT is counted only once in the PT; the worst CTCAE grade is counted. ^*^ Peripheral sensory neuropathy.								

### Efficacy

In the glioblastoma cohort, one patient had a confirmed partial response with a steady decrease in target lesion size to less than 10% over a treatment period of 16 cycles (see Online Resource 1). Another patient with stable disease as best response was treated for 10 cycles, corresponding to a disease control rate of 2/8 (25%) after eight cycles of treatment, and an objective response rate of 1/8 (12.5%) in the EEP of this cohort. In the ovarian cancer cohort, the best response was stable disease in three patients, corresponding to disease control rates of 3/8 (37.5%) after two cycles of treatment in the EEP. After four cycles, the disease control rate was 1/8 (12.5%) in the EEP. Reduction in the size of target lesions was observed in four patients with ovarian cancer and in three patients with glioblastoma.

**Fig. 1:**
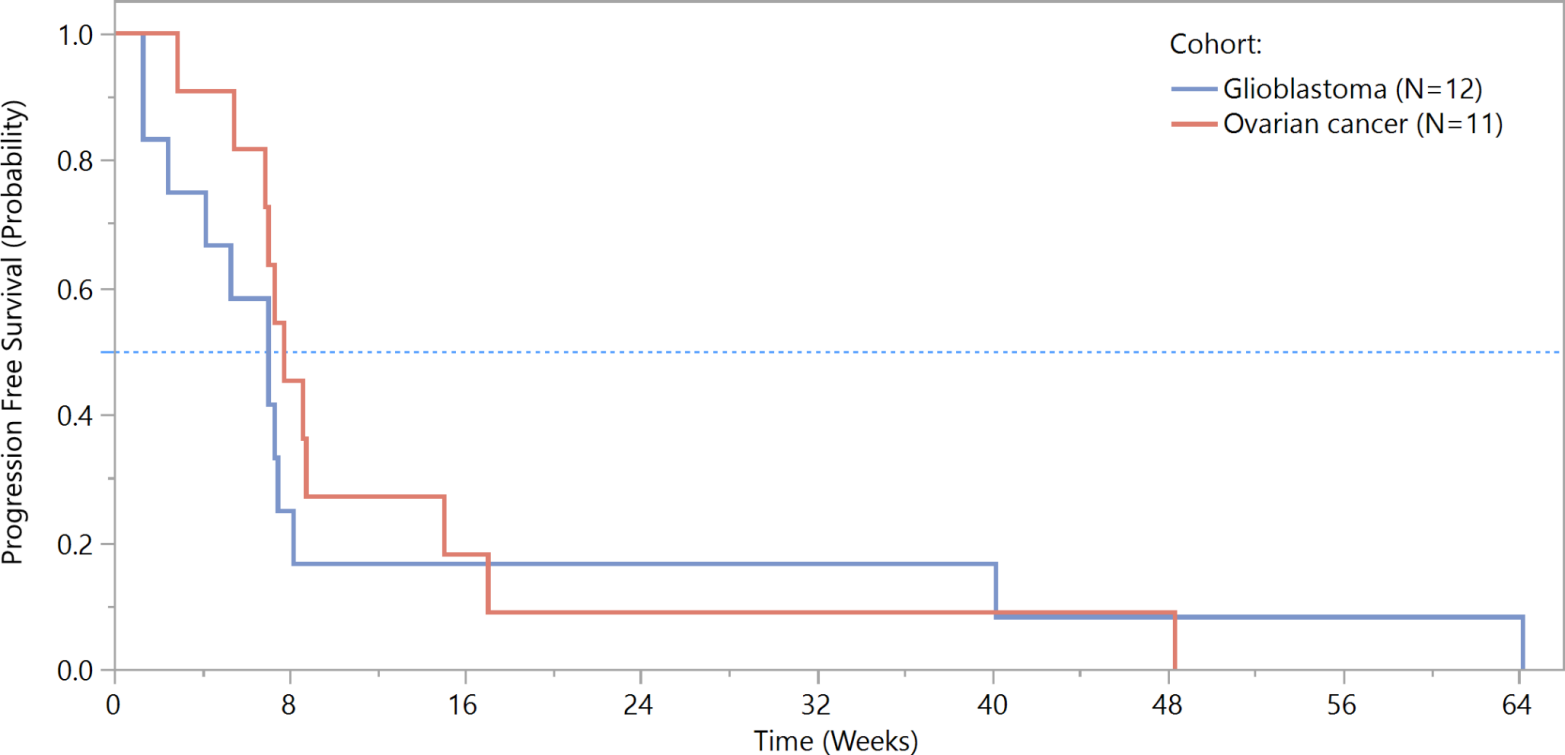
Progression-free survival (full analysis population)

All study patients experienced disease progression. The six-month PFS rates in the ovarian cancer and glioblastoma cohorts were 9.1% and 16.7%, respectively. Kaplan-Meier curves for PFS are shown in Fig. 1. The median time to disease progression was similar in both cohorts (54.0 and 49.0 days for the ovarian cancer and glioblastoma cohorts, respectively). In the overall EEP, the six-month PFS rates were 12.5% and 25%, with median times to death or disease progression of 55.5 and 51.5 days in the ovarian cancer and glioblastoma cohorts, respectively.

## Discussion

Lisavanbulin is a water-soluble lysine prodrug of the active synthetic small molecule avanbulin, which can be administered intravenously in the absence of solubilizing excipients, and has high oral bioavailability. Study CDI-CS-001 showed that the RP2D of 30 mg/m^2^ was well-tolerated and had encouraging anti-tumor activity [13]. However, the observed dose-limiting toxicities of lisavanbulin were related to its effects on the vascular system, which occurred at peak serum concentrations (C_max_), whereas its anti-proliferative effects were related to drug exposure (AUC) [13]. The goal of this study was therefore to test the safety, tolerability, and anti-tumor efficacy of lisavanbulin using a 48-hour IV infusion.

When administered as a 48-hour IV infusion, the RP2D was determined to be 70 mg/m^2^ in the Phase 1 portion of this study [14]. Administration of lisavanbulin in this manner resulted in a higher dose intensity and higher cumulative exposure to the active moiety avanbulin, while mitigating the vascular toxicity observed with the 2-hour infusion [14]. The corresponding mean C_max_ values were lower with the 48-hour infusion (144 vs. 267 ng/mL for the 2-hour infusion), resulting in a four-fold higher AUC/C_max_ ratio (60 vs. 14, respectively) [14]. In the Phase 1 portion of the study, dose-limiting toxicities comprised grade 3 hypotension in one patient treated at 70 mg/m^2^, grade 3 hyponatremia in one patient at 90 mg/m^2^, and grade 2 hallucination, ataxia, and dysarthria with grade 3 neutropenia in another patient treated at 90 mg/m^2^ [14]. The only corresponding event of the same grade during the Phase 2a portion of the study was a case of grade 2 hallucination leading to dose reduction in a patient with ovarian cancer. Grade 3/4 events were reported in three patients with ovarian cancer and in one with glioblastoma. Two patients experienced grade 3 hypertension; one patient with pre-existing hypertension had self-limiting grade 3 episodes in cycle 1; another patient with hypertension borderline to grade 2 at baseline required initiation of antihypertensive medication in cycle 12. The interpretation of one event of grade 4 decrease in lymphocyte counts requires some caution, as it only occurred 12 days after the last infusion had ended; such events were rarely observed with lisavanbulin, and when seen were of low grades. The case of grade 3 increase in aspartate aminotransferase would be classified as grade 1 per CTCAE version 5.0 due to elevated baseline values. Overall, the safety profile of lisavanbulin appears consistent in patients with solid tumors, ovarian cancer, and glioblastoma treated at the RP2D. When comparing the percentage of patients affected by related AEs (89%, 81%, and 33%; Table 2), the tolerance to lisavanbulin appears to slightly favor patients with glioblastoma. Treatment compliance was high in both Phase 2a cohorts. Compared with study CDI-CS-001, the percentages of patients affected by related adverse events appear similar across system organ classes at RP2D levels (CDI-CS-001: 30 mg/m^2^, CDI-CS-003: 70 mg/m^2^) but for gastrointestinal, nervous system, and vascular disorders, these percentages were significantly lower than those observed in study CDI-CS-001 at dose levels of 45–80 mg/m^2^ (see Online Resource 3).

Signals of efficacy were observed following treatment with lisavanbulin as a 48-hour IV infusion. In the ovarian cancer cohort, three patients had best response of stable disease, with lesion size reductions after two cycles of treatment (EEP). In the glioblastoma cohort, one patient with partial response was treated for 16 cycles, and one patient with stable disease as best response was treated for 10 cycles, corresponding to a disease control rate of 25% after eight cycles of treatment, and an objective response rate of 12.5% in the EEP.

In a Phase 1 study with continuous daily oral administration of lisavanbulin, two of 28 patients with recurrent or progressive glioblastoma or high-grade glioma achieved long-lasting objective responses (CDI-CS-002, NCT02490800 [15]). Notably, the tumor tissues of both patients showed strong end-binding protein 1 (EB1) expression as assessed by immunohistochemistry staining. This observation is supported by earlier data in orthotopic glioblastoma mouse models suggesting that EB1 expression could be a response-predictive biomarker of lisavanbulin in glioblastoma [17, 18]. The rather low prevalence of high EB1 expression seen in archival tissue samples of various tumor types [19] could explain heterogeneous responses seen with lisavanbulin when used in unselected patient populations. The Phase 2a portion of study CDI-CS-002 was therefore designed to determine the objective response rate of daily oral administration of lisavanbulin (25 mg per day) in patients whose glioblastoma tissue had high EB1 expression [20]. While oral administration has obvious advantages, data generated in this study support the safe use of lisavanbulin as a 48-hour continuous infusion, which may gain additional significance if response-predictive biomarkers of lisavanbulin show high prevalence in pediatric indications.

## Conclusion

This study demonstrated that a 48-hour continuous IV infusion of lisavanbulin had a better tolerability profile than the 2-hour infusion. The favorable safety profile was confirmed for patients with solid tumors, including glioblastoma.

## Electronic supplementary material

Below is the link to the electronic supplementary material.

Supplementary Material 1

Supplementary Material 2

Supplementary Material 3
