# Supplementary material for: Safety and anti-tumor activity of lisavanbulin administered as 48-hour infusion in patients with ovarian cancer or recurrent glioblastoma: a phase 2a study
Source: Invest New Drugs. 2023 Feb 16;41(2):267–75. doi: 10.1007/s10637-023-01336-9 (PMC10140113; doi:10.1007/s10637-023-01336-9)

Electronic supplementary material

Article title: Safety and anti-tumor activity of lisavanbulin administered as 48-hour infusion in patients with ovarian cancer or recurrent glioblastoma: A Phase 2a study

Journal: Investigational New Drugs

Authors: Markus Joerger, Thomas Hundsberger, Simon Haefliger, Roger von Moos, Andreas F. Hottinger, Thomas Kaindl, Marc Engelhardt, Michalina Marszewska, Heidi Lane, Patrick Roth, Anastasios Stathis.

Corresponding author: Thomas Kaindl, MD. Basilea Pharmaceutica International Ltd, Allschwil, Hegenheimermattweg 167b, 4123 Allschwil, Switzerland; Tel: +41 (0)61 567 1505;

E-Mail: Thomas.Kaindl@basilea.com

Online resource 1: MRIs of the glioblastoma patient with partial response and > 90% change from baseline in cycle 10

Baseline

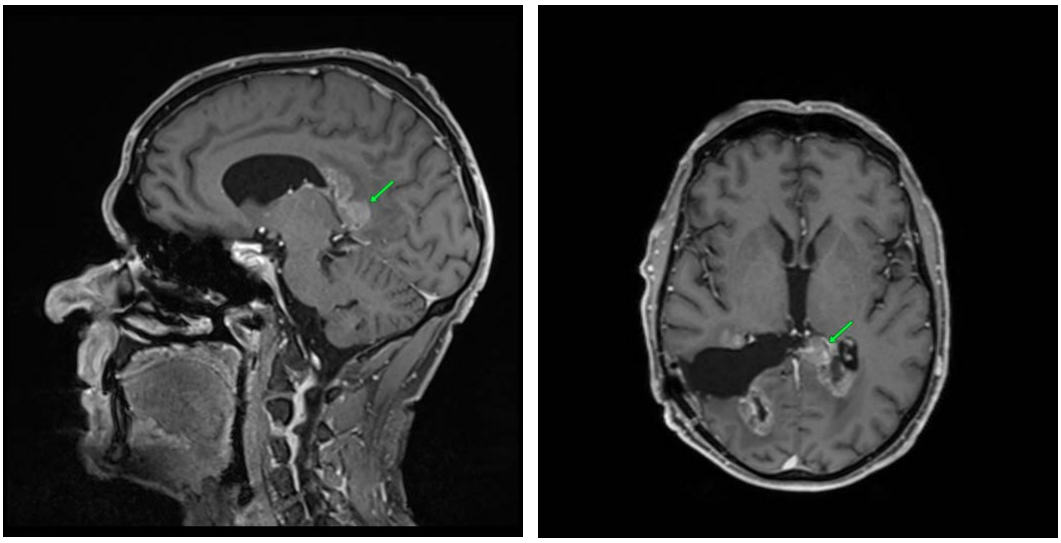

Cycle 10

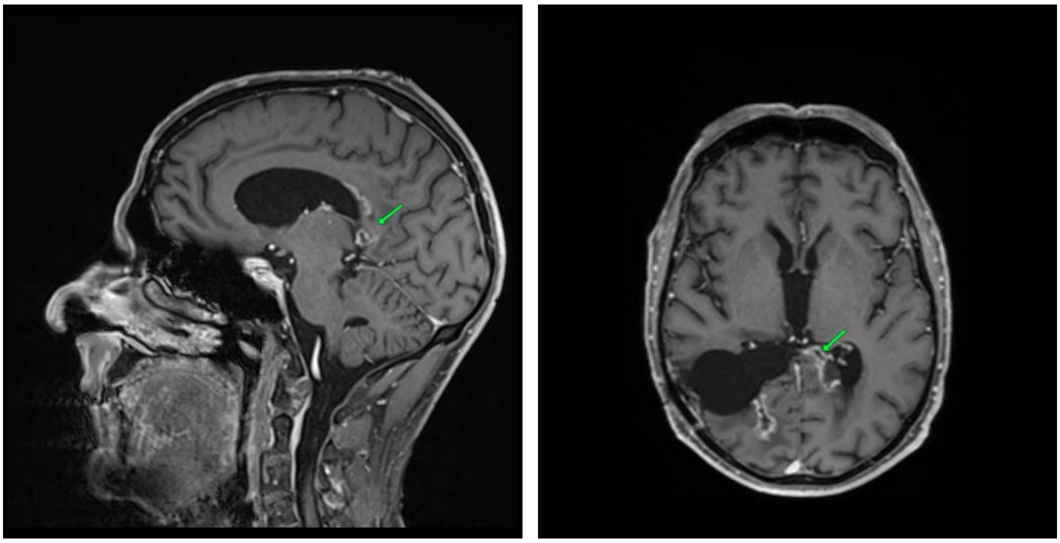

Supplement: Supplementary file 1 — Supplementary Material 1 [file 10637_2023_1336_MOESM1_ESM.pdf]
