# Supplementary material for: Safety and anti-tumor activity of lisavanbulin administered as 48-hour infusion in patients with ovarian cancer or recurrent glioblastoma: a phase 2a study
Source: Invest New Drugs. 2023 Feb 16;41(2):267–75. doi: 10.1007/s10637-023-01336-9 (PMC10140113; doi:10.1007/s10637-023-01336-9)
Supplement: Supplementary file 2 — Supplementary Material 2 [file 10637_2023_1336_MOESM2_ESM.pdf]

Electronic supplementary material

Article title: Safety and anti-tumor activity of lisavanbulin administered as 48-hour infusion in patients with ovarian cancer or recurrent glioblastoma: A Phase 2a study

Journal: Investigational New Drugs

Authors: Markus Joerger, Thomas Hundsberger, Simon Haefliger, Roger von Moos, Andreas F. Hottinger, Thomas Kaindl, Marc Engelhardt, Michalina Marszewska, Heidi Lane, Patrick Roth, Anastasios Stathis.

Corresponding author: Thomas Kaindl, MD. Basilea Pharmaceutica International Ltd, Allschwil, Hegenheimermattweg 167b, 4123 Allschwil, Switzerland; Tel: +41 (0)61 567 1505;

E-Mail: Thomas.Kaindl@basilea.com

Online Resource 2: Most common treatment-emergent adverse events by system organ class and preferred term in the Phase 2a safety population (> 10% of patients)

System Organ Class (SOC)<br>Preferred Term (PT)	Cohort; n (%)		
	**Ovarian cancer**	**GBM**	**Overall**
**Safety population**	**n = 11**	**n = 12**	**n = 23**
Number of Patients with at Least one AE	11 (100%)	11 (91.7%)	22 (95.7%)
Nervous system disorders	5 (45.5%)	11 (91.7%)	16 (69.6%)
Paraesthesia	3 (27.3%)	2 (16.7%)	5 (21.7%)
Headache	0	3 (25.0%)	3 (13.0%)
Gastrointestinal disorders	9 (81.8%)	6 (50.0%)	15 (65.2%)
Constipation	6 (54.5%)	2 (16.7%)	8 (34.8%)
Abdominal pain	6 (54.5%)	0	6 (26.1%)
Nausea	1 (9.1%)	4 (33.3%)	5 (21.7%)
Abdominal pain upper	2 (18.2%)	1 (8.3%)	3 (13.0%)
Diarrhea	3 (27.3%)	0	3 (13.0%)
Vomiting	2 (18.2%)	1 (8.3%)	3 (13.0%)
General disorders and administration site conditions	6 (54.5%)	6 (50.0%)	12 (52.2%)
Fatigue	6 (54.5%)	4 (33.3%)	10 (43.5%)
Oedema peripheral	2 (18.2%)	1 (8.3%)	3 (13.0%)
Psychiatric disorders	6 (54.5%)	5 (41.7%)	11 (47.8%)
Insomnia	4 (36.4%)	0	4 (17.4%)
Musculoskeletal and connective tissue disorders	8 (72.7%)	2 (16.7%)	10 (43.5%)
Muscle spasms	3 (27.3%)	2 (16.7%)	5 (21.7%)
Back pain	4 (36.4%)	0	4 (17.4%)
Metabolism and nutrition disorders	7 (63.6%)	2 (16.7%)	9 (39.1%)
Decreased appetite	6 (54.5%)	1 (8.3%)	7 (30.4%)
Hypokalaemia	3 (27.3%)	1 (8.3%)	4 (17.4%)
Respiratory, thoracic and mediastinal disorders	6 (54.5%)	2 (16.7%)	8 (34.8%)
Dyspnea	4 (36.4%)	1 (8.3%)	5 (21.7%)
Vascular disorders	5 (45.5%)	2 (16.7%)	7 (30.4%)
Hypertension	3 (27.3%)	0	3 (13.0%)
Infections and infestations	2 (18.2%)	4 (33.3%)	6 (26.1%)
Investigations	3 (27.3%)	2 (16.7%)	5 (21.7%)
Blood and lymphatic system disorders	3 (27.3%)	1 (8.3%)	4 (17.4%)
Anaemia	3 (27.3%)	1 (8.3%)	4 (17.4%)
Eye disorders	1 (9.1%)	2 (16.7%)	3 (13.0%)
Injury, poisoning and procedural complications	1 (9.1%)	2 (16.7%)	3 (13.0%)
Fall	1 (9.1%)	2 (16.7%)	3 (13.0%)

A patient with multiple events within a SOC/PT was counted only once within the SOC/PT, respectively. SOCs and PTs were coded from the Medical Dictionary for Regulatory Activities (MedDRA) Version 19.0.
GBM, glioblastoma.
