# Supplementary material for: Safety and anti-tumor activity of lisavanbulin administered as 48-hour infusion in patients with ovarian cancer or recurrent glioblastoma: a phase 2a study
Source: Invest New Drugs. 2023 Feb 16;41(2):267–75. doi: 10.1007/s10637-023-01336-9 (PMC10140113; doi:10.1007/s10637-023-01336-9)
Supplement: Supplementary file 3 — Supplementary Material 3 [file 10637_2023_1336_MOESM3_ESM.pdf]

Electronic supplementary material

Article title: Safety and anti-tumor activity of lisavanbulin administered as 48-hour infusion in patients with ovarian cancer or recurrent glioblastoma: A Phase 2a study

Journal: Investigational New Drugs

Authors: Markus Joerger, Thomas Hundsberger, Simon Haefliger, Roger von Moos, Andreas F. Hottinger, Thomas Kaindl, Marc Engelhardt, Michalina Marszewska, Heidi Lane, Patrick Roth, Anastasios Stathis.

Corresponding author: Thomas Kaindl, MD. Basilea Pharmaceutica International Ltd, Allschwil, Hegenheimermattweg 167b, 4123 Allschwil, Switzerland; Tel: +41 (0)61 567 1505;

E-Mail: Thomas.Kaindl@basilea.com

Online resource 3: Treatment-emergent related adverse events by system organ class; safety population treated in studies CDI-CS-001 and CDI-CS-003 at the recommended Phase 2 dose (RP2D) and higher dose levels

	**48h-infusion (CDI-CS-003), n (%)**				**2h-infusion (CDI-CS-001), n (%)**			
	**70 mg/m² (RP2D) (N = 32)**		**90 mg/m² (N = 4)**		**30 mg/m² (RP2D) (N = 36)**		**45–80 mg/m² (N = 36)**	
**System Organ Class (SOC)**	Any grade	Grade ≥3	Any grade	Grade ≥3	Any grade	Grade ≥3	Any grade	Grade ≥3
Gastrointestinal disorders	8 (25)	0	3 (75)	0	18 (50)	0	30 (83) + *	2 (6)
General disorders and administration site conditions	10 (31)	0	3 (75)	0	13 (36)	1 (3)	21 (58)	2 (6)
Nervous system disorders	7 (22)	1 (3)	1 (25)	0	8 (22)	0	22 (61) + *	2 (6)
Vascular disorders	3 (9)	3 (9)	0	0	4 (11)	1 (3)	22 (61) + *	17 (47) + *
Metabolism and nutrition disorders	9 (28)	0	3 (75)	1 (25)	4 (11)	0	10 (28)	1 (3)
Musculoskeletal and connective tissue disorders	4 (13)	0	2 (50)	0	0	0	10 (28) *	2 (6)
Investigations	3 (9)	2 (6)	2 (50)	1 (25)	8 (22)	0	1 (3)	1 (3)
Psychiatric disorders	4 (13)	0	2 (50)	0	0	0	4 (11)	1 (3)
Skin and subcutaneous tissue disorders	1 (3)	0	1 (25)	0	4 (11)	0	4 (11)	0
Blood and lymphatic system disorders	0	0	0	0	6 (17)	0	2 (6)	0
Respiratory, thoracic and mediastinal disorders	2 (6)	0	0	0	1 (3)	0	3 (8)	1 (3)
Cardiac disorders	0	0	0	0	1 (3)	0	4 (11)	2 (6)
Neoplasms benign, malignant and unspecified	0	0	0	0	0	0	4 (11)	1 (3)
Eye disorders	1 (3)	0	0	0	0	0	1 (3)	0
Infections and infestations	1 (3)	0	0	0	1 (3)	0	0	0
Injury, poisoning and procedural complications	1 (3)	0	0	0	0	0	1 (3)	0

If a patient had more than one event within a primary SOC, the patient was counted only once

Fisher's exact test of patients affected by related events of any grade / grade ≥ 3:
+ $p < 0.01$ compared with 70 mg/m² 48h-infusion
* $p < 0.01$ compared with 30 mg/m² 2h-infusion
